# Near-infrared-light pre-treatment attenuates noise-induced hearing loss in mice

**DOI:** 10.7717/peerj.9384

**Published:** 2020-06-17

**Authors:** Dietmar Basta, Moritz Gröschel, Ira Strübing, Patrick Boyle, Felix Fröhlich, Arne Ernst, Rainer Seidl

**Affiliations:** 1 Department of ENT at ukb, Charité Medical School, University of Berlin, Berlin, Germany; 2 Advanced Bionics GmbH, Hanover, Germany

**Keywords:** Near infrared light, Noise-induced hearing loss, Auditory brainstem response, Cochlear hair cells

## Abstract

Noise induced hearing loss (NIHL) is accompanied by a reduction of cochlear hair cells and spiral ganglion neurons. Different approaches have been applied to prevent noise induced apoptosis / necrosis. Physical intervention is one technique currently under investigation. Specific wavelengths within the near-infrared light (NIR)-spectrum are known to influence cytochrome-c-oxidase activity, which leads in turn to a decrease in apoptotic mechanisms. It has been shown recently that NIR can significantly decrease the cochlear hair cell loss if applied daily for 12 days after a noise exposure. However, it is still unclear if a single NIR-treatment, just before a noise exposure, could induce similar protective effects. Therefore, the present study was conducted to investigate the effect of a single NIR-pre-treatment aimed at preventing or limiting NIHL. The cochleae of adult NMRI-mice were pre-treated with NIR-light (808 nm, 120 mW) for 5, 10, 20, 30 or 40 minutes via the external ear canal. All animals were noised exposed immediately after the pre-treatment by broad band noise (5–20 kHz) for 30 minutes at 115 dB SPL. Frequency specific ABR-recordings to determine auditory threshold shift were carried out before the pre-treatment and two weeks after the noise exposure. The amplitude increase for wave IV and cochlear hair cell loss were determined. A further group of similar mice was noise exposed only and served as a control for the NIR pre-exposed groups. Two weeks after noise exposure, the ABR threshold shifts of NIR-treated animals were significantly lower (*p* < 0.05) than those of the control animals. The significance was at three frequencies for the 5-minute pre-treatment group and across the entire frequency range for all other treatment groups. Due to NIR light, the amplitude of wave four deteriorates significantly less after noise exposure than in controls. The NIR pre-treatment had no effect on the loss of outer hair cells, which was just as high with or without NIR-light pre-exposure. Relative to the entire number of outer hair cells across the whole cochlea, outer hair cell loss was rather negligible. No inner hair cell loss whatever was detected. Our results suggest that a single NIR pre-treatment induces a very effective protection of cochlear structures from noise exposure. Pre-exposure of 10 min seems to emerge as the optimal dosage for our experimental setup. A saturated effect occurred with higher dosage-treatments. These results are relevant for protection of residual hearing in otoneurosurgery such as cochlear implantation.

## Introduction

Noise affects the morphology and function of peripheral and central auditory structures. Different mechanisms lead to damage to inner and outer cochlear hair cells, e.g., mechanical intervention, ischemia (Ohgami et al., 2012; Gyo, 2013; Moussavi Najarkola et al., 2013; Perny et al., 2017), oxidative stress (Evans & Halliwell, 1999; Henderson et al., 2006) or calcium dysregulation (Le Prell et al., 2006). Microscopic signs for outer hair cells’ damage include: uncoupling from the tectorial membrane and mechanical disruption of their stereociliary arrays (Liberman & Beil, 1979; Slepecky, 1986; Patuzzi, Yates & Johnstone, 1989; Nordmann, Bohne & Harding, 2000; Kurabi et al., 2017). Such processes are initiated very rapidly following noise exposure (Lin et al., 2011; Bullen et al., 2019) and can lead in turn to irreversible loss of outer hair cells and their mechanical amplifying abilities (Wagner et al., 2005; Henderson et al., 2006; Salvi et al., 2017). Severe to profound hearing loss is largely the result of an inner hair cell loss. However, frequently a reduced innervation of inner hair cells appears to be responsible for the extent of the noise-induced hearing loss, evidenced by spiral ganglion cells dying after noise exposure, even when the structure of the inner hair cells obviously survives (Kujawa & Liberman, 2009; Choi & Choi, 2015).

Various pharmacological approaches have been undertaken to prevent noise-induced hearing loss. The most promising strategies appear to be: anti-inflammatory therapies, use of antioxidants as inhibitors of intracellular stress pathways, neurotrophic factors, inhibition of programmed cell death pathways, and neurotransmission blockers. For an overview see Waqas et al. (2018). Unfortunately, most of these interventions carry undesired side effects. A quite successful neuroprotective approach, without serious side-effects, appears to be the use of near-infrared (NIR) light. NIR-light has already been applied in various fields of medicine (Hashmi et al., 2010). It has regulatory approval for the treatment humans having skin diseases (630 nm, Eells et al., 2004; Martins, Trindade & Leite, 2008), pain (Chow et al., 2009) and stroke (808 nm, Lampl et al., 2007).

Several in vitro and in vivo studies have investigated the effects of NIR-treatment in the auditory system in response to cochlear injury. In vitro studies have demonstrated protection of cochlear or vestibular hair cell cultures after pharmacological insult: e.g., gentamycin-induced damage (Rhee et al., 2012b; Chang et al., 2019). Additionally, in vivo studies were able to show protective effects, mediated by near-infrared light, for both aminoglycoside ototoxicity (Rhee et al., 2013; Zhang et al., 2015; Lee et al., 2017) and noise-induced hearing loss (Tamura et al., 2015; Tamura et al., 2016; Rhee et al., 2012a; Lee et al., 2016a).

Animal experiments into noise exposure have shown a decreased cochlear hair cell loss and a significantly lower hearing threshold shift for a daily NIR-treatment over 12 days following a trauma induced by noise exposure (Rhee et al., 2012a). The underlying physiological mechanism of NIR-light effects is called “photo-biomodulation”, triggered by light in the red to near-infrared range (630 to 1,000 nm). Specific wavelengths within the spectrum of NIR-light are known to influence cytochrome-C-oxidase activity via photon absorption, which leads in turn to a decrease of apoptotic mechanisms (Wong-Riley et al., 2005). Cytochrome-C-oxidase is part of the mitochondrial respiratory chain with absorption maxima of 680 and 830 nm, where cytochrome C is oxidized and molecular oxygen is reduced to water. This chain transports protons directly from mitochondria, maintaining the membrane potential for an activation of the adjusted ATP synthase, hence leading to a highly significant increase in ATP. High levels of ATP are known to reduce cell apoptosis (Oron et al., 2007). The ATP increase leads primarily to an enhancement of the cAMP level, calcium release and gene expression (Wong-Riley et al., 2001; Byrnes et al., 2005b; Liang et al., 2006). The numerous important outcomes of this triggered cascade are: decreased neuroinflammation (Byrnes et al., 2005a), reduced expression of the proapoptotic protein Bax, increased expression of the anti-apoptotic protein Bcl-2 (Liang et al., 2006), decreased amounts of superoxide radicals and nitric oxide production (Liang et al., 2008).

From in vitro studies in the auditory periphery, a strong correlation has been found between NIR-treatment, following gentamicin application, and mitochondrial function, accompanied by elevated ATP levels and an increased mitochondrial membrane potential (Chang et al., 2019). This idea is also supported by in vivo NIR experiments showing the activation of superoxide dismuthase-1 (SOD-1), a mitochondria-related enzyme with antioxidative properties, in the vestibular sensory epithelium (Zhang et al., 2015). Although most studies in the inner ear focused on the protective properties of NIR-treatment on sensory tissue, that is cochlear and vestibular hair cells, recent investigations have also observed neuroprotective effects in the cochlea. Studies by Lee and colleagues observed NIR-related rescue of neural structures, including inner hair cell postsynaptic puncta, neurofilaments and spiral ganglion cells, when applied after ouabain treatment, a drug leading to auditory neuropathy by selectively damaging spiral ganglion cells (Lee et al., 2016b). In another study, the same group was also able to demonstrate a protection from inner hair cell synaptopathy through NIR treatment following moderate noise exposure (Lee et al., 2019; Chang et al., 2019).

A decreased amount of superoxide radicals and nitric oxide (NO) production was observed upon a single 100 s NIR pre-treatment of cultured outer hair cells before gentamicin application, compared to gentamicin application alone (Bartos et al., 2016). Superoxide radicals react with NO and form peroxynitrite, which blocks cellular respiration and diminishes ATP production (Srinivasan & Avadhani, 2012). To our knowledge no in vivo studies have yet been conducted to assess the impact of NIR-treatment applied before auditory insult (i.e., a pre-treatment to induce protective effects). Our hypothesis is that an increase of the intracellular ATP-level by an NIR-pre-treatment would pre-condition for cochlear neuroprotection during a subsequent trauma. Therefore, the present study’s rationale is to investigate the effect of a single NIR-pre-treatment on hearing and hair cell loss following noise exposure made immediately after the pre-treatment.

## Material and Methods

### Auditory brainstem responses

Frequency-specific auditory brainstem responses (ABR) were recorded for 5 kHz spaced frequencies, between 5 and 40 kHz inclusive, in adult female mice of the NMRI strain (Charles River Laboratories, Erkrath, Germany). The mice were aged 10 to 11 weeks. All mice were caged in groups of 6 animals. In addition to a resting house, each cage contained enrichment features (plastic tunnels and nesting material). The animals had permanent access to food and water and were kept in a 12/12 h dark/light regime. All mice were divided among the experimental groups so that no animal survived. The study protocol stipulated that animals with a weight loss of more than 20% should be euthanatized by a single injection of 180 mg/kg ketamine and 18 mg/kg xylazine. No animal reached this criterion during the study.

**Figure 1:**
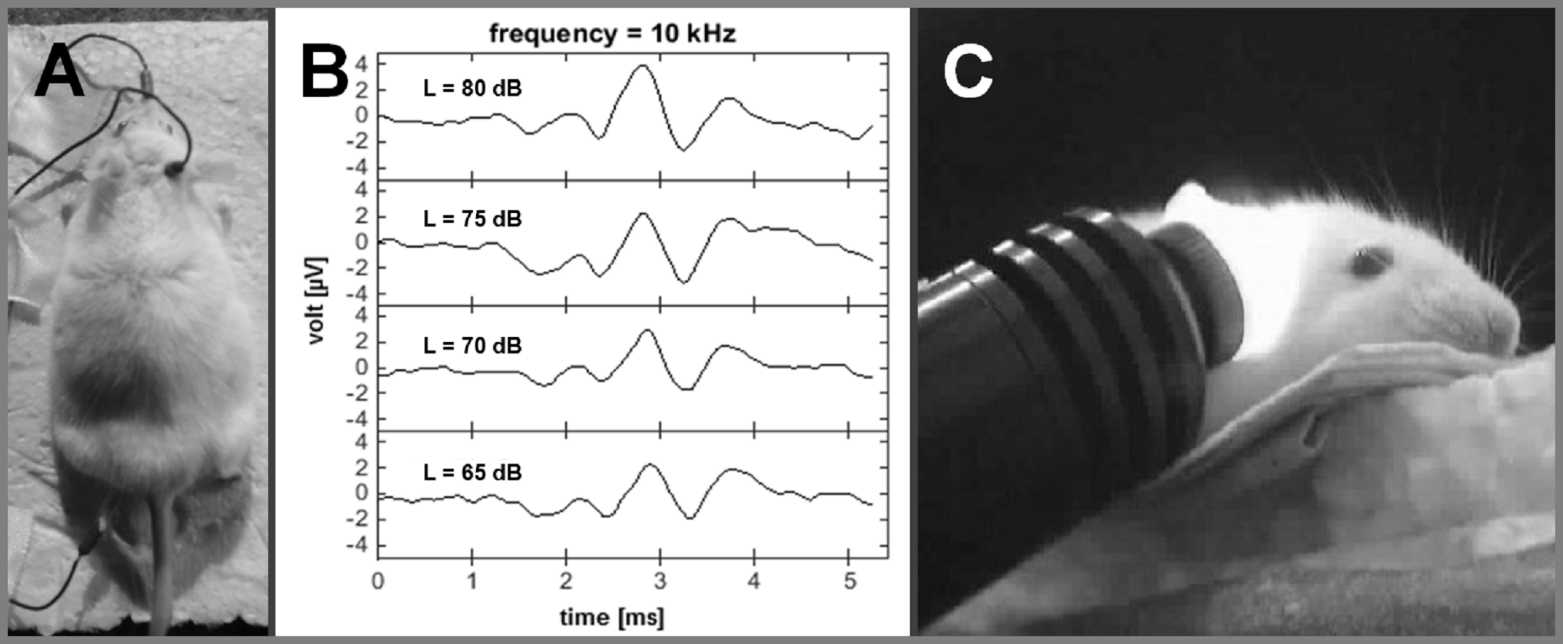
Auditory brainstem recordings. Frequency specific ABR-recordings before and after NIR-light-treatment (electrode positions: mastoid = recording; nose = reference; foot = ground; A); ABR-waves II, III and IV at a frequency of 10 kHz and different sound levels (65–80 dB SPL; B); NIR-light beam fully cover the mouse cochlea via the outer ear canal with specific angle for a NIR-light pre-treatment (visualized by a NIR-camera; C).

ABR recordings were made under anaesthesia (60 mg/kg ketamine, 6 mg/kg xylazine) two days before and two weeks after a noise exposure. Tone stimuli were presented binaurally at different sound pressure levels (SPL) using a sine-wave generator (FG 250 D, H-Tronic, Germany) and were adjusted with an audio amplifier (AMP-50, Tangent, Denmark). ABR waveforms were collected from the output of a recording amplifier (USB-ME16-FAI-System, Multi-Channel Systems, Germany). Subdermal needle electrodes were placed (Fig. 1A) on the forehead (reference), mastoid (active) and at one foot (ground). For each individual frequency, the peak-to-peak amplitudes within the maximum deflection of wave III/IV were measured for different sound intensities (Fig. 1B). The ABR threshold per frequency was estimated by visual inspection of the series of recordings for each frequency and determining when the wave III/IV was no longer visible.

From these data, threshold differences for each frequency (mean threshold shifts) were calculated between the controls and the noise-exposed animals using the group average values. Results are represented as mean relative hearing loss (±S.E.) in dB for each experimental group compared to the control group.

### NIR pre-treatment

Two control groups were formed, one without any noise exposure or NIR pre-treatment (control; *n* = 7) and one with noise-exposure but without NIR-treatment (noise only; *n* = 16). The remaining animals were unilaterally treated with NIR-light immediately before noise exposure of different durations, depending on the experimental group: 5 min NIR-treatment (5′ NIR; *n* = 16), 10 min NIR-treatment (10 ′NIR; *n* = 16), 20 min NIR-treatment (20 ′NIR; *n* = 7), 30 min NIR-treatment (30 ′NIR; *n* = 8), 40 min NIR-treatment (40 ′NIR; *n* = 8). An adjustable isolated point laser module (DB808-120-3(22 ×65), Picotronic, Germany) was used for the application of near-infrared light having a wavelength of 808nm and a power of 120mW. The laser module was placed at the outer ear canal at exactly the angle that allowed a total coverage of the cochlea with the laser beam of approximately seven mm diameter (Fig. 1C). The power density of the NIR-light was 312 mW/cm^2^. Safety in terms of tissue damage in general, and to the tympanic membrane in particular, has been verified by several other studies using higher power densities. For example Rhee et al. (2013) used a power density of 900 mW/cm^2^, delivered for 60 min/day over10 days). Moreover, Moon et al. (2016) demonstrated tympanic membrane damage resulting from NIR application for power densities above at least 1,137 mW/cm^2^ applied for 30 minutes/day over 14 consecutive days. This previous work dispelled any safety concerns for the NIR power density applied in the present study (Moon et al., 2016).

### Noise exposure

Just after receiving its NIR pre-treatment, each animal was exposed for 30 min to noise in a soundproof chamber measuring 0.8 m × 0.8 m × 0.8 m, having a minimal sound attenuation of 60 dB). Noise exposure was made using a broad-band white noise with corner frequencies of 5 and 20 kHz and having a level of 115 dB SPL. Noise was delivered binaurally from a loudspeaker (HTC 11.19, Visaton, Germany) placed above the animal’s head. The speaker was connected to an audio amplifier, which provided the broad-band noise from a DVD source (DK DVD-438, DK, Germany). The noise level was calibrated by using a sound level meter (Voltcraft 329, Voltcraft, Germany) placed close to the animal’s ear. Anaesthesia was controlled through observation via a video camera inside of the lighted chamber. Body temperature was maintained at a constant level of 37 °C using a heating pad.

### Histological analysis

After its post-noise exposure ABR recording, each animal was perfused via the left heart chamber with 4% paraformaldehyde (PFA, pH 7.4, Sigma, Germany) in order to fixate the neural tissue. Inner ears were carefully removed from the skull and stored in PFA at 4 °C until further processing. Both cochleae of each animal were prepared for staining. After decalcification of the cochleae for 8 h with the solution renewed after 4 h, using 0.4 M ethylenediaminetetraacetic acid (EDTA, pH 8.0, Roth, Germany), cochleae were dissected twice horizontally to obtain large segments of half turns. Half turns were stained by using the Alexa-fluor-488-phalloidin staining to visualise F-actin compartments in the cytoskeleton. Cochleae were placed in a 24-well plate and washed with 2 ml phosphate buffered saline (PBS, Gibco, USA). Two ml of 0.2% Triton-X (Sigma, Germany) in PBS was added to the cochleae as permeabilization solution and incubated for 30 min on a shaker at room temperature. After washing with 2 ml PBS, 80 µl Alexa-Fluor-488-phalloidin dilution (1:40 in PBS, Molecular Probes, USA) was added and incubated for 60 min on a shaker at room temperature in the dark. All cochlear segments were embedded with RotiMount Fluorocare and diamidino phenylindole (DAPI, Roth, Germany) on microscope slides with coverslips over night at room temperature in the dark.

The stained cochleae were magnified microscopically with fluorescence channels for phalloidin (480 nm) and DAPI (340 nm). The images were digitized with the AxioCam ICc1 (Zeiss, Germany) and the ZEN 2.3 camera software (Zeiss, Germany). The number of missing hair cells was counted by hand on printouts of overlaid DAPI and phalloidin staining pictures. The gaps between cells were marked using a pen so that the number of cells that were missing could be counted.

The basilar membrane’s length was estimated using the ImageJ 1.47d software (National Institutes of Health, USA).

### Statistical procedures

The hearing threshold data and cochlear hair cell counts were analysed and compared between the experimental and the control groups by comparing the results using the *U*-test (not normally distributed data) or the *t*-test (normally distributed data). Data distribution was tested using the Kolmogoroff-Smirnoff-test. The SPSS software (IBM SPSS Statistics Version 25, IBM Corp., USA) was used for all statistical analyses. The level of significance for all statistical tests was set at *p* < 0.05. A Bonferroni alpha correction was applied for the use of multiple comparisons.

The Landesamt für Gesundheit und Soziales, Berlin, Germany approved the study protocol (approval number LAGeSo-G 0146/14).

## Results

### Effect on ABR-threshold shift

**Figure 2:**
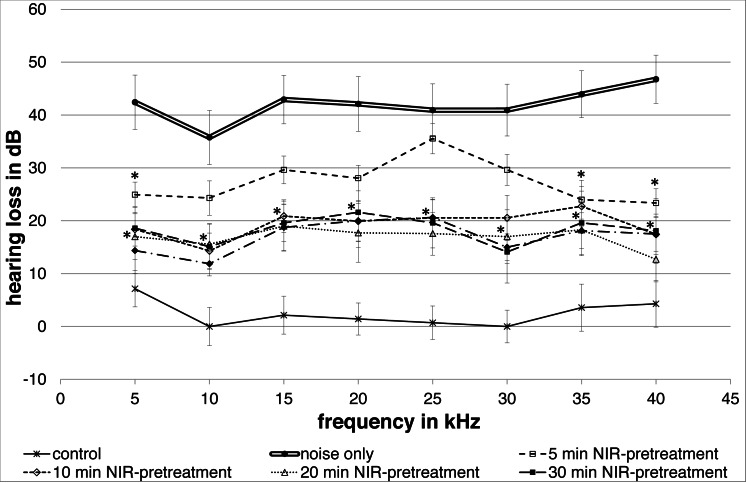
Noise induced hearing loss. Means (±SE) of noise-induced hearing loss for different NIR-light pre-treatments of the cochlea. The 5-minute pre-treatment group shows statistically significant differences at 5, 35 and 40 kHz. All other pre-treated groups show statistically significant differences at all frequencies. The control group without noise and NIR pre-treatment showed only a negligible hearing loss. Filled asterisks indicate statistically significant differences between the pre-treatment groups and the “noise only”-group.

All NIR pre-treated groups showed a lower group mean hearing loss following the noise exposure compared to the “noise only”-control group (Fig. 2). A frequency-specific analysis showed significant changes between the controls and the 5′NIR-group for the 5; 35 and 40 kHz frequencies. All other pre-treated groups differed significantly from the noise only controls for every frequency tested (Fig. 2). No statistically significant differences between ABR-thresholds were found for the 10′; 20′; 30′and 40′ NIR-groups. The “control”-group showed only a small, negligible hearing loss.

### Effect on amplitude increase of ABR wave IV

**Figure 3:**
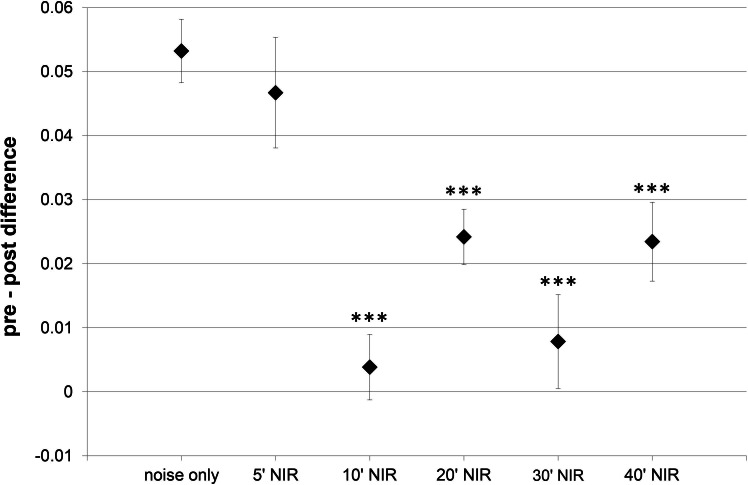
Slope within the amplitude growth function of ABR wave IV. Difference of the slope of the linear part within the amplitude growth function of ABR wave IV (averaged over all tested frequencies) between pre- and post-exposure measurements (post-noise exposure values are subtracted from pre-exposure values). A higher slope difference indicates a smaller post-treatment slope. Asterisks mark statistically significant differences between the NIR-treated groups and the “noise only” -group.

Due to the ABR recording electrode positions, ABR-wave IV was the only wave that was robust and prominent in all animals and experimental groups. This is why more detailed ABR-analysis was limited to this wave. Additional wave IV analysis aimed to identify any alteration in central suprathreshold processing resulting from noise exposure, with or without NIR pre-treatment. Wave IV represents bilateral activation of the auditory midbrain level, hence any changes in stimulus-related responses could point to modified central auditory processing. The slope of the wave IV amplitude growth function’s linear part was calculated and the post-exposure slope was subtracted from the pre-exposure slope (Fig. 3). The average slope difference over all frequencies tested for the “10′-NIR”-group (slope difference 0.004  ±  0.005 µV/dB) was more than 10 times lower than for the “noise only”-group (slope difference 0.053 ± 0.005 µV/dB). A higher slope difference indicates a smaller post-noise exposure slope for the amplitude growth function.

Apart from the values of the “5′-NIR”-group (0.047 ± 0.009 µV/dB) all experimental groups showed significantly lower slope differences compared to the “noise-only”-group (Fig. 3).

### Effect on cochlear hair cells

**Figure 4:**
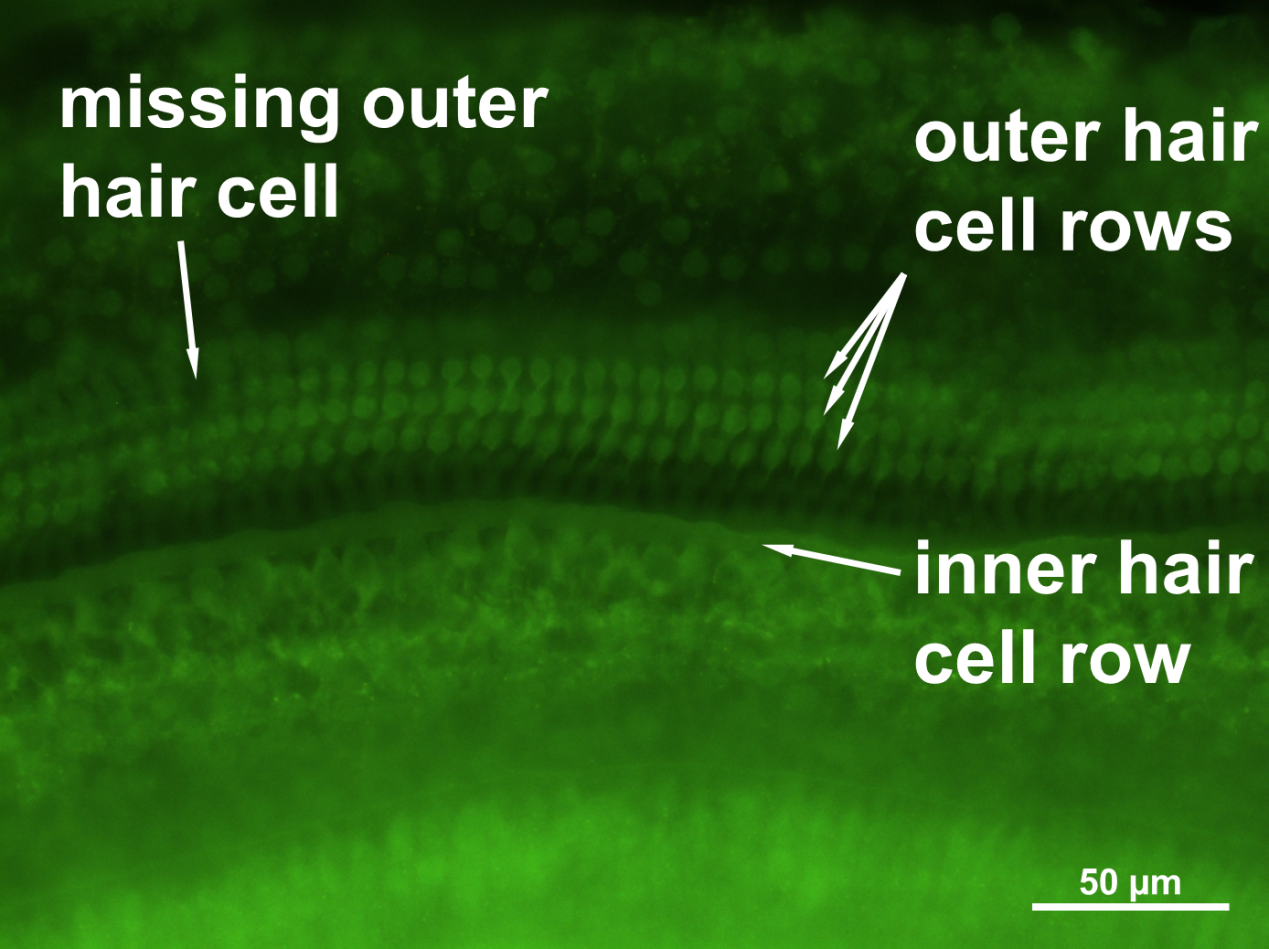
Cochlear hair cell preparation. Sample microphotograph with scale bar of the mouse cochlear whole mount immunofluorescence staining (400×  magnification).

**Figure 5:**
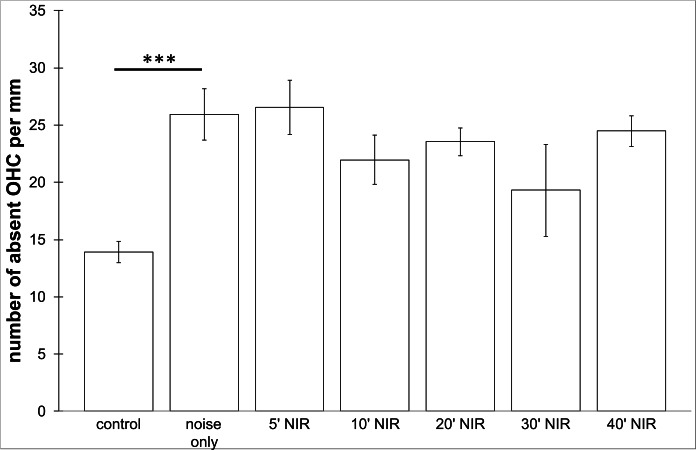
Outer hair cell counting. Means (±SE) of absent outer hair cells per mm on the pre-treated side following different duration of NIR-light exposure. Asterisks indicate statistically significant differences. The “control” -group was not noise exposed and received no NIR pre-treatment. The “noise only” -group received no NIR pre-treatment.

**Figure 6:**
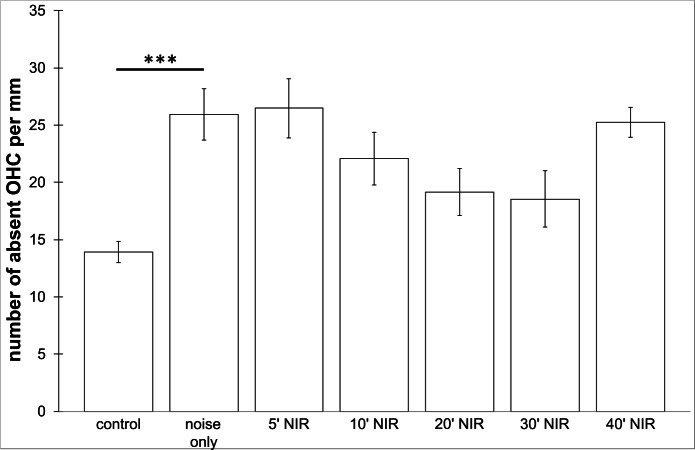
Outer hair cell counting. Means (±SE) of absent outer hair cells per mm on the not pre-treated side following different duration of contra-lateral NIR-light exposure. Asterisks indicate statistically significant differences. The “control” -group was not noise exposed and received no NIR pre-treatment. The “noise only” -group received no NIR pre-treatment.

**Figure 7:**
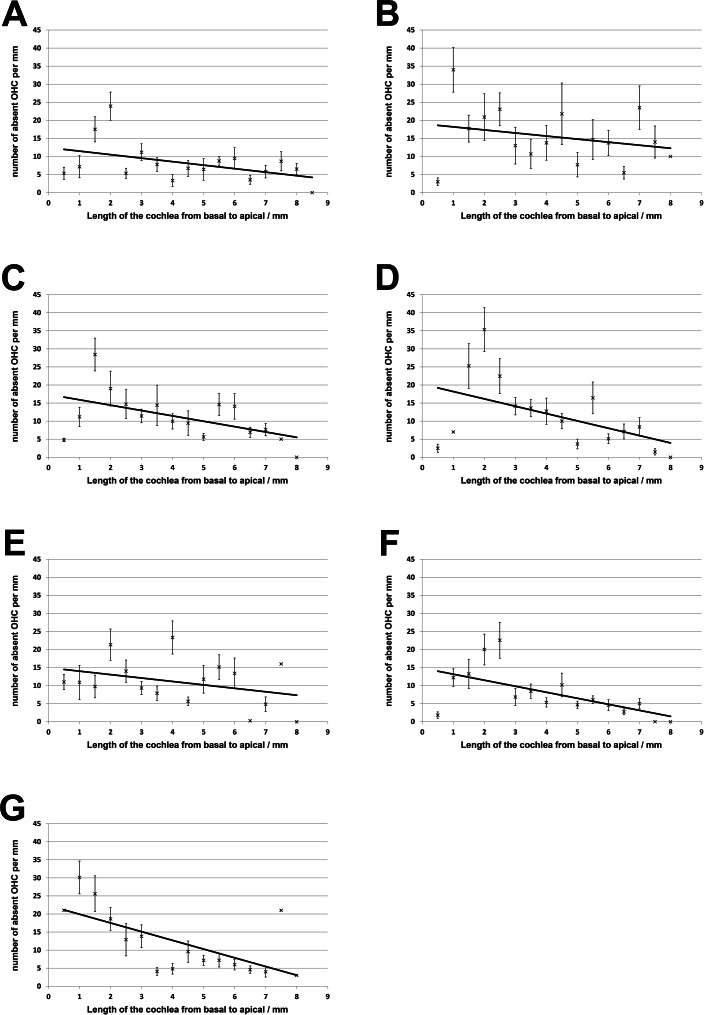
Distribution of cochlear outer hair cell loss. Distribution of outer hair cell loss along the organ of corti for all experimental and control groups (A, untreated controls; B, noise only; C, 5 min NIR-pretreatment; D, 10 min NIR-pretreatment; E, 20 min NIR-pretreatment; F, 30 min NIR-pretreatment; G, 40 min NIR-pretreatment). Error bars represent standard error.

No loss of inner hair cells could be detected bilaterally for any of the groups. Figure 4 shows a sample image of the whole cochlear immunofluorescence staining mount. For the noise only control group, a significant decrease in outer hair cell counts was found for both ears (Figs. 5 and 6), compared to the control group where no noise exposure was given. However, the percentage of missing outer hair cells was only 6.5% compared to a normal cochlea (Burda, Ballast & Bruns, 1988). No significant differences were found between the number of absent outer hair cells in any of the NIR pre-treated experimental groups and the “noise only”-group (Fig. 5). This also holds true for the non-pre-treated side (ear) of the animals (Fig. 6). In addition, the mean number of absent outer hair cells was plotted for the entire length of the organ of corti (Fig. 7). These graphs indicate that hair cell loss decreased from base to apex without any specific correlation to the functional analysis (ABR-thresholds). This was found for all experimental and control groups.

## Discussion

The present results show that a single NIR pre-treatment of at least 10 min produces a significantly protective effect on the entire frequency range that we investigated: 5 to 40 kHz. The protective effect plateaued when a longer NIR pre-treatment was applied. It is hypothesized that this is related to a saturation effect for ATP generation. A pre-treatment of 5 min showed a statistically significant protection only at low (5 kHz) and high (35 and 40 kHz) frequencies. The area in between these frequencies corresponds well with the broad-band noise trauma applied. It appears that the activation of protective mechanisms produced by the shorter pre-treatment was too weak for the main noise-affected frequency range, but sufficient for the surrounding areas.

### Efficiency of NIR pre-treatment

The noise-induced elevation of hearing thresholds, as determined by ABR-measurements, was reduced on average by 24 dB in the NIR-pre-treated groups when calculated across all effective treatment groups and the whole frequency range investigated. This shows that a single pre-treatment has a similar effect to a daily post-traumatic NIR-treatment delivered over 12 consecutive days (Rhee et al., 2012a). However, the energy density of the NIR-treatment in the present study was twice as that used by Rhee et al. (2012a). It should also worth considering that the exposure time in the earlier investigation was 12 ×  60 min (total of 720 min), instead of a single 10 min treatment in the present study. Thus, the total NIR-dosage applied in the post-treatment experiments was much higher than for this pre-treatment study. Another consideration is that the noise exposure differed greatly between the two studies: 115 dB SPL broad-band white noise (5–20 kHz) for 30 min in this study, compared to a 116 dB SPL narrow band noise (centred at 16 kHz (bandwidth 1 kHz) for 6 h in the post-treatment study. However, the effect on hearing thresholds was quite similar for both studies, showing a threshold shift of around 40 dB. This could be related to the different species used in both studies (rats and mice) and their possibly different susceptibility to noise.

Thus, a pre-treatment regime appears to be much more effective than several post-treatments. The reason for this is largely unknown. Earlier studies have shown that pre-conditioning using a very low NIR dose induced a significant decrease in superoxide radicals and NO production for cultured outer hair cells (Bartos et al., 2016). This could have resulted in an enhanced intracellular ATP level compared to the non-pre-treated cells, since superoxide radicals react with NO to form peroxynitrite, which blocks cellular respiration (Srinivasan & Avadhani, 2012). Furthermore, recent studies have revealed that the nuclear factor NF- *κ*B is activated by NIR-treatment. This leads to protection against inducible NO synthase-triggered oxidative stress and, subsequently, to a reduced caspase-3-mediated apoptosis (Tamura et al., 2016). The time point of NIR-treatment plays an important role in the activation or inactivation of NF- κB. The difference being the presence or absence of inflammation in the cells at the time of the treatment (Chen et al., 2011). Pro inflammatory cytokines are activated during a noise exposure (Nakamoto et al., 2012). Thus, the effects of NIR pre-treatment are not affected by these inflammatory processes. This could explain why NIR pre-treatment is much more efficient than post-traumatic treatment.

### Effect on cochlear hair cells

Apart from the quite similar outcome found for hearing preservation in our study, there are some differences in outer hair cell counts compared to earlier investigations. Former studies described a significant reduction of outer hair cells and no loss of inner hair cells due to noise exposure (Rhee et al., 2012a; Tamura et al., 2015). These reports are in line with our present results. However, NIR post-treatment led to a significant reduction of outer hair cell loss in the medial part of the cochlea. The basal and apical parts showed no statistically significant protective effect from NIR-light on hair cell loss (Rhee et al., 2012a). Another study which applied the NIR treatment after noise exposure found a protection of outer hair cells in all parts of the cochlea (Tamura et al., 2015). However, both studies applied a more intense noise trauma than in the present investigation. The relative loss of outer hair cells was very small in the present study compared to the earlier work: 6.5% vs approx. 30% (Tamura et al., 2015). Hence, a significant rescue of outer hair cells was harder to achieve in the present study. The present results show a trend for hair cell protection, even if no correlation between hair cell loss along the cochlea and frequency specific ABR-thresholds could be found. The bilateral effect is not contradictory for this hypothesis since both sides (left and right cochleae) are likely simultaneously treated due to the surprisingly good penetration of NIR-light of 808 nm through the small mouse skull (Lee et al., 2016a). Additionally, the discrepancy between hair cell counts and hearing thresholds, as found in the present study, is also well known for both animals and humans (Le Calvez et al., 1998; Landegger, Psaltis & Stankovic, 2016). The correlation coefficient between hearing threshold shift and cochlear hair cell loss was described as only around 0.5 with 1.0 being a perfect correlation (Landegger, Psaltis & Stankovic, 2016).

It is possible that functional damage to hair cells occurred in the present experiments (e.g., stereocilia Wang, Hirose & Liberman, 2002), or that other cochlear structures are responsible for the hearing loss and the NIR-related preservation observed. Suitable candidates which are known to be influenced by NIR-light are spiral ganglion cells, pre- and postsynaptic structures at the inner hair cells (ribbon synapse), cochlear neurofilaments and fibrocytes of the lateral wall (Tamura et al., 2016; Lee et al., 2016b; Lee et al., 2019; Chang et al., 2019). A first sign of such effects could be the relationship between the amplitude of a summating potential, which is generated within the auditory brainstem/midbrain (e.g., ABR wave IV), and the stimulus intensity. The significant differences that we describe between the slopes of this relationship in animals with and without a NIR-pre-treatment supports this hypothesis. The lower slope difference in NIR pre-treated animals indicates a near-normal sensitivity to an increased stimulus intensity and thus a near-normal loudness growth. This could be related to a preservation of cochlear structures by NIR-light.

## Conclusions

In essence, the present results show that a single NIR pre-treatment of at least 10 min produces a significant hearing protection upon noise exposure. The observed effect was not based on the protection of cochlear hair cell density.

Further investigations are necessary to identify the anatomical correlates for the functional preservation of hearing resulting from a single NIR pre-treatment.

##  Supplemental Information

10.7717/peerj.9384/supp-1 Supplemental Information 1 Auditory brainstem response thresholds Each data point indicates the frequency specific auditory brainstem response threshold of the individual animal before and after noise exposure.

10.7717/peerj.9384/supp-2 Supplemental Information 2 Auditory brainstem response wave IV amplitude growth function The table show the amplitude growth functions of auditory brainstem response wave IV and the seperated slope of the function for each measurement.

10.7717/peerj.9384/supp-3 Supplemental Information 3 Counting of missing cochlear hair cells Missing cochlear hair cells per mm and cochlear length measurements.

## Additional Information and Declarations

### Data Availability

The following information was supplied regarding data availability: All raw measurements are available in the Supplementary Files.
